# Iridoschisis: à propos d’un cas

**DOI:** 10.11604/pamj.2016.24.242.7460

**Published:** 2016-07-15

**Authors:** Mouhoub Imane, Sekhsoukh Rachid

**Affiliations:** 1 Service d’Ophtalmologie, CHU Mohammed VI, Oujda, Maroc

**Keywords:** Iridoschisis, glaucome, cataracte, Iridoschisis, glaucoma, cataract

## Image en medicine

L’iridoschisis est une pathologie dégénérative rare au cours de laquelle le stroma de l’iris se sépare en deux couches, un feuillet antérieur qui se fend en fibrilles qui fluctuent dans l’humeur aqueuse, et un feuillet postérieur qui reste attaché au muscle dilatateur et à l’épithélium pigmenté rétinien. Il touche préférentiellement les personnes âgées, et est souvent compliqué d’un glaucome par fermeture de l’angle, une cataracte et d’une kératopathie bulleuse. L’image montre un patient de 74 ans, qui consulte pour une baisse de l’acuité visuelle. L’examen ophtalmologique montre (A) une cornée transparente, une chambre antérieure peu profonde, une pupille ronde et centrée, une atrophie de l’iris et une fragmentation du feuillet stromal plus importante du coté gauche avec une cataracte totale blanche de l’œil gauche. La gonioscopie (B) objective un angle irido-cornéen peu visible en inférieur gêné par les débris iriens flottants et étroit sur le reste. L’examen UBM (C) montre un clivage de l'iris en deux couches. Le patient a bénéficié d’une cure de cataracte avec bonne amélioration de l’acuité visuelle (D).

**Figure 1:**
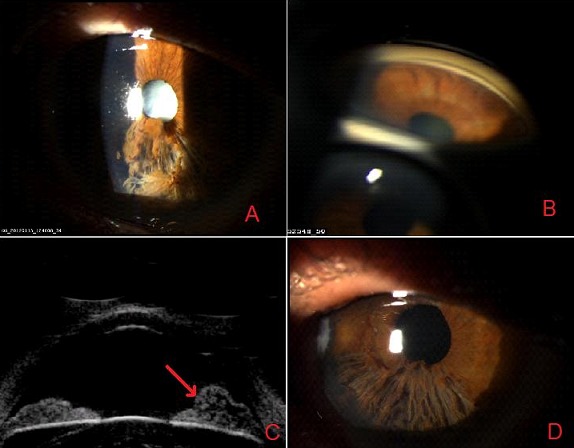
(A) dégénérescence irienne inférieure; (B) angle irido-cornéen étroit; (C) UBM clivage de l’iris en deux couches; (D): aspect après cure de cataracte

